# Extremely high troponin levels induced by septic shock: a case report

**DOI:** 10.1186/s13256-021-03027-6

**Published:** 2021-09-11

**Authors:** Naoki Matsunaga, Yuki Yoshioka, Yasushi Fukuta

**Affiliations:** Department of Emergency and Critical Care Medicine, Tokushima Red Cross Hospital, 103, Irinokuchi, Komatsushima-cho, Komatsushima City, Tokushima 773-8502 Japan

**Keywords:** Sepsis, Sepsis-induced cardiomyopathy, Sepsis-induced myocardial dysfunction, Septic cardiomyopathy, Troponin

## Abstract

**Background:**

Troponin levels can be elevated in various diseases other than acute myocardial infarction, including sepsis. In diseases without myocardial necrosis, the elevated troponin levels are relatively low and normalize quickly.

**Case presentation:**

A 61-year-old Japanese man with impaired consciousness was transported to our hospital. He was diagnosed as having pneumonia and septic shock. His condition was severe, but his clinical course was good. However, his troponin level remained extremely high during admission; on the second day, it was higher than the measurable range. We consulted a cardiologist and performed echocardiography and myocardial perfusion scintigraphy but found no new ischemic changes.

**Conclusion:**

In septic shock, troponin levels can be extremely high, which can persist even after recovery, as in very large myocardial infarctions.

## Introduction

Troponin level is elevated in various diseases other than acute myocardial infarction (AMI), including sepsis. In these diseases, the elevated troponin level is relatively low; the higher the troponin, the greater the possibility of AMI [1]. Moreover, in AMI, the elevated troponin level persists for 7–10 days owing to necrotic release from cardiomyocytes, but in other diseases, the troponin level declines relatively rapidly [1]. A report showed that patients with sepsis who had higher troponin levels tended to have a higher mortality rate [2]. Herein, we report a case of septic shock in which the troponin level was extremely high and persisted even after recovery from sepsis, despite the absence of myocardial necrosis.

## Case report

**Fig. 1:**
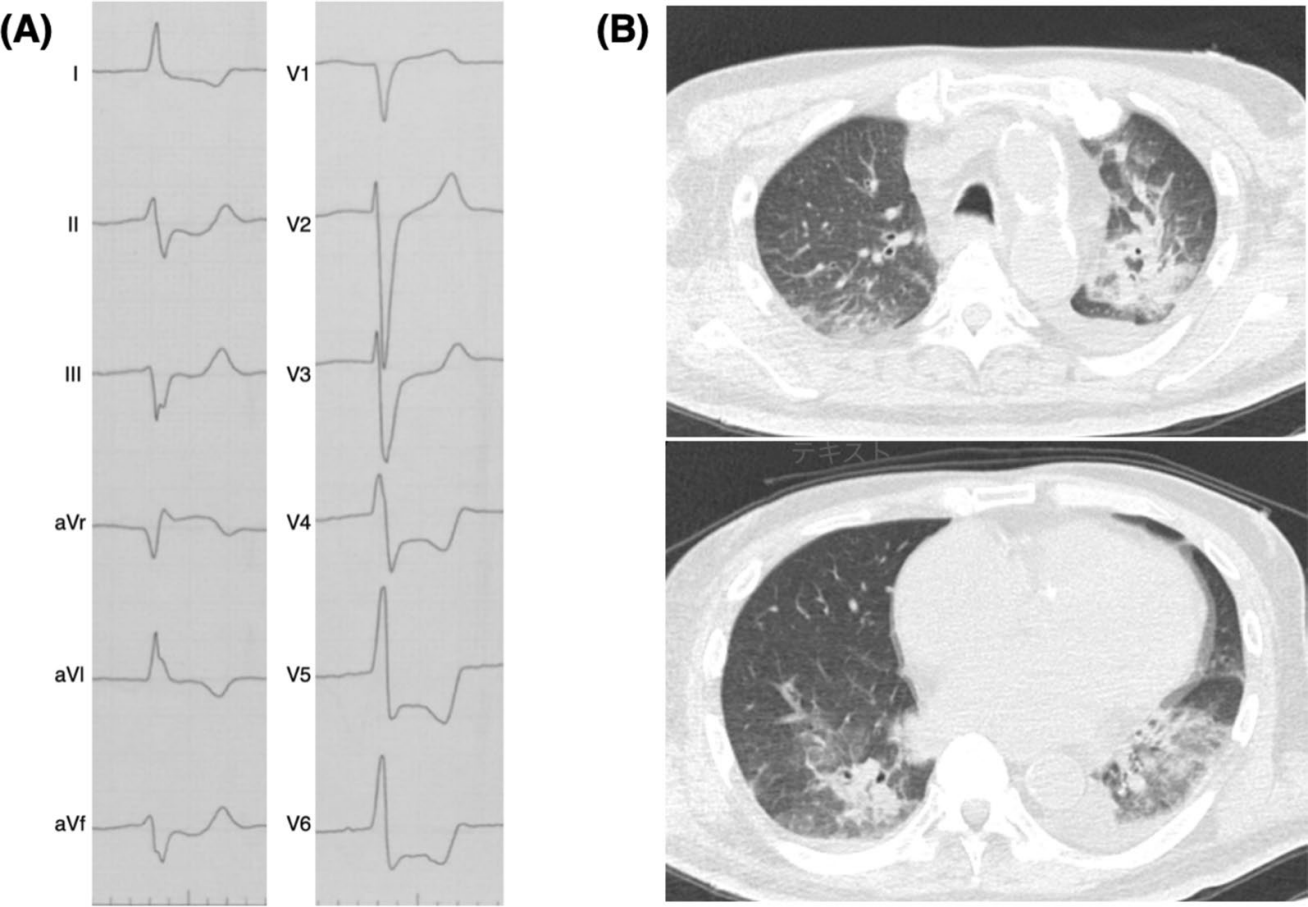
**A** Electrocardiogram on admission showing ST depression in leads I, II, III, aVL, aVF, and V3–V6, with ST elevation in the aVR lead. **B** Chest computed tomography image on admission showing consolidations in the right lower, left upper, and lower lobes

A 61-year-old Japanese man with impaired consciousness was transported to our hospital. He had a history of type 2 diabetes mellitus, old myocardial infarction, angina pectoris, and chronic kidney disease (CKD). On initial evaluation, he had shock with cool peripheries, blood pressure of 79/68 mmHg, heart rate of 78 beats per minute, Glasgow Coma Scale score of 3/15, and axillary temperature of 31 °C. Additionally, transthoracic echocardiography (TTE) revealed diffuse left ventricular wall dyskinesia, with a visual ejection fraction (EF) of 20–30% (originally 47% with posterolateral wall hypokinesia/akinesia). Electrocardiography revealed widespread ST depression with ST elevation in the aVR lead (Fig. 1A). Laboratory findings showed leukocytosis, acute kidney injury (AKI), metabolic acidosis, anemia, hypoglycemia, and elevated high-sensitivity cardiac troponin I (hs-cTnI) level (Table 1). Chest computed tomography revealed bilateral consolidation (Fig. 1B). We consulted a cardiologist because of the extremely high hs-cTnI level (54,138 ng/L), but the cardiologist considered AMI unlikely. The patient was diagnosed as having pneumonia (sputum culture grew *Streptococcus pneumoniae*) and septic shock, and acute upper gastrointestinal bleeding. The Acute Physiology and Chronic Health Evaluation II and Sequential Organ Failure Assessment scores were 54 and 12, respectively. In the emergency department, orotracheal intubation was performed, and antimicrobial administration, fluid resuscitation, vasopressor agent administration, and blood transfusion were started. After admission to the intensive care unit, continuous hemodiafiltration (CHDF) was also started for AKI. On the first day of admission, he had a pulseless electrical activity (PEA) twice, but the return of spontaneous circulation was achieved at both times with a 1 g adrenaline dose. CHDF was terminated on the second day, and vasopressors/inotropic agent administration was terminated on the third day. The patient was weaned off from the ventilator on the seventh day and transferred to the hospital for rehabilitation on the 23rd day. After the transfer, he had no problems and was discharged.

**Table 1:** Blood test results at admission

White blood cell count (/pL)	24,710
Hemoglobin (g/dL)	5.9
Creatinine (mg/dL)	3.61 (originally 1.9)
Potassium (mEq/L)	7.1
pH	6.814
Base excess (mmol/L)	−30.9
Anion gap (mmol/L)	29.9
Lactate (mmol/L)	16.18
Blood glucose (mg/dL)	50
hs-cTnI* (ng/L)	54,138 (reference range < 27)

*high-sensitivity cardiac troponin I

**Fig. 2:**
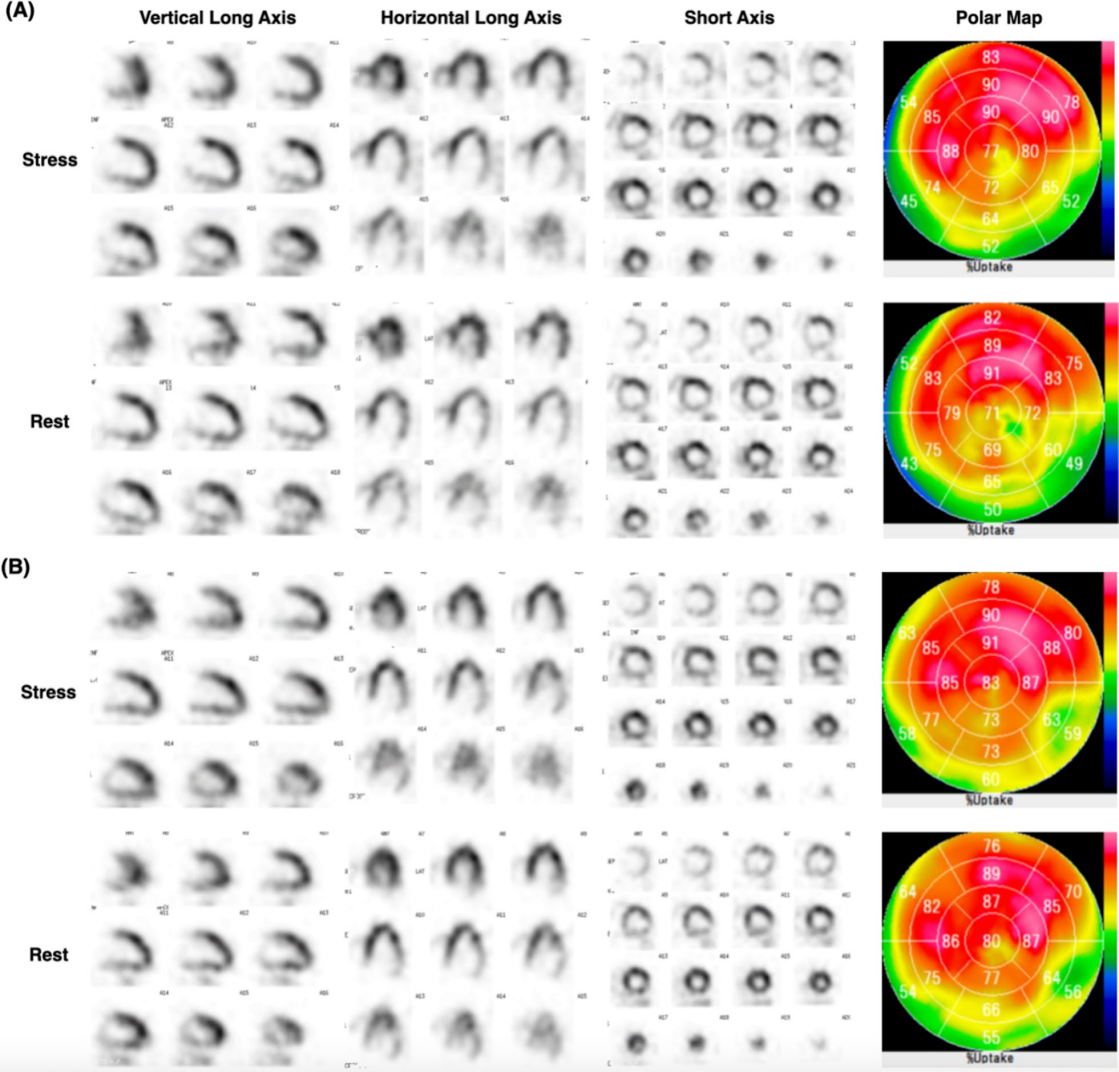
Myocardial perfusion scintigraphy image showing no evidence of new infarction. **A** Myocardial perfusion scintigraphy images on the 21st day of hospitalization and **B** before admission

Nevertheless, his hs-cTnI level continued to be extremely high during admission. It was > 500,000, 193,309, 29,357, and 4747 ng/L on the second, third, 13th, and 20th days, respectively. On the 17th day, TTE was performed, no new asynergy was found, and the EF improved to 38%. On the 21st day, myocardial perfusion scintigraphy was performed under the care of a cardiologist, and no new myocardial necrosis was observed (Fig. 2).

## Discussion

We found that sepsis can cause an extremely high troponin level, as in very large myocardial infarctions. No previous studies have reported that troponin levels in sepsis can be as high as in this case. A study that used the same troponin assay that we used (ARCHITECT STAT hs-TnI, Abbott) showed that the median hs-cTnI levels (ng/L) in sepsis on the first through the third day were 109 [interquartile range (IQR) 39–394], 103 (IQR 38–449), and 79 (IQR 31–281), respectively [3]. In the present case, the patient’s hs-cTnI level was higher than the measurable range (> 500,000 ng/L), and we suspected that the measurement might be inaccurate. However, the hs-cTnI level on the next day was also extremely high (193,309 ng/L); thus, we thought that the measurement of the troponin levels was accurate. As for the complication of AMI, we consulted a cardiologist and even performed myocardial scintigraphy; consequently, no new ischemic changes occurred. In addition, takotsubo cardiomyopathy, myocarditis, and pulmonary embolism are also causes of very high troponin levels [1], but no findings were suggestive of these diseases. Therefore, we concluded that the extremely high hs-cTnI levels were due to sepsis-induced myocardial injury.

In this case, the high troponin level persisted for at least 20 days. In general, troponin levels decline relatively quickly in diseases other than AMI [1]. However, a previous report also showed that, in some cases of sepsis, troponin levels remained elevated even on the seventh day [4]. Hence, high troponin levels are likely to persist in sepsis.

The present case had a good outcome even though the troponin level continued to be extremely high. A recent study showed that, in sepsis, the higher the hs-cTnI level, the higher the mortality rate, but the relationship disappeared when the level was > 100–500 ng/L [3]. Therefore, the extremely high troponin level in sepsis may not indicate a poor prognosis in proportion to the value. In addition, we consider that septic cardiomyopathy (SCM) may have contributed to the troponin elevation and favorable outcome in our case. No clear diagnostic criteria have been established for SCM, but the characteristic echocardiographic findings are left ventricular dilatation and decreased EF, both of which are reversible [5]. These findings were also observed in our case, which indicated that the patient had SCM. A report showed that, in sepsis with SCM, troponin levels were around four times higher than in sepsis without SCM [6]. The prognosis in SCM with hypokinetic conditions (EF < 40%, no tachycardia, large left ventricle, and so on) is considered good [7].

SCM alone cannot explain why the troponin level was extremely elevated in our case. The patient had a history of coronary artery disease and CKD. These are factors that cause elevation of troponin level in sepsis [3]. Moreover, he had complications of anemia and hypoglycemia, and PEA on the first day. These etiologies can also cause elevation of troponin level [8–10]. The elevation of troponin level, in this case, is thought to be caused by a combination of SCM and these conditions.

## Conclusion

Herein, we report a case of extremely high hs-cTnI levels induced by septic shock. In spite of the high levels, the patient’s clinical outcome was good, and no evidence of new myocardial ischemic findings was found.

## Data Availability

The data for this case report are located at Tokushima Red Cross Hospital.
